# Supplementary material for: Barriers to Healthcare Access During the Coronavirus Disease 2019 (COVID-19) Pandemic: A Cross-Sectional Study Among Romanian Patients with Chronic Illnesses and Confirmed SARS-CoV-2 Infection
Source: Healthcare (Basel). 2025 Jun 3;13(11):1333. doi: 10.3390/healthcare13111333 (PMC12155512; doi:10.3390/healthcare13111333)
Supplement: Supplementary file 1 [file healthcare-13-01333-s001.zip › healthcare-3615482-supplementary.pdf]

SATISFACTION QUESTIONNAIRE

Supplementary Material S1 – Full English Translation of the Original Questionnaire Used in the Study
"Barriers to Healthcare Access During the Coronavirus Disease 2019 (COVID-19) Pandemic: A Cross-Sectional Study Among Romanian Patients with Chronic Illnesses and Confirmed SARS-CoV-2 Infection."

Note: This is the full English version of the questionnaire that was originally administered in Romanian to study participants. It has been translated faithfully and item-by-item, as applied in the research.

I. SOCIOECONOMIC CONDITIONS

1.You are:

❑ A patient diagnosed with at least one of the five chronic conditions under investigation and who has also contracted the SARS-CoV-2 infection
❑ A caregiver of a patient over 18 years old diagnosed with at least one of the five chronic conditions under investigation and who has also contracted the SARS-CoV-2 infection

2.Your age:

❑ Under 20 years old
❑ Between 20 and 30 years old
❑ Between 30 and 40 years old
❑ Between 40 and 50 years old
❑ Between 50 and 60 years old
❑ Over 60 years old
❑ Over 80 years old

3.Your gender:

❑ Female
❑ Male

4.Highest level of education completed:

❑ Middle school
❑ High school
❑ Vocational school
❑ Post-secondary school
❑ Bachelor's degree
❑ Master's degree
❑ PhD

5.Residence:

- ❑ Rural
- ❑ Urban

6.What is your monthly income?

- ❑ Below 2000 RON
- ❑ Between 2000 and 3000 RON
- ❑ Between 3000 and 4000 RON
- ❑ Between 4000 and 5000 RON
- ❑ Above 5000 RON

II. PATIENT HISTORY

7.Have you been diagnosed with a chronic illness?

- ❑ YES
- ❑ NO

8.Do you suffer from one of the following five chronic conditions where the addition of asystemic infection such as SARS-CoV-2 may result in more severe disease?

- ❑ Ischemic Heart Disease (IHD)
- ❑ Arterial Hypertension (HTN)
- ❑ Neoplasms (Cancer)
- ❑ Chronic Obstructive Pulmonary Disease (COPD)
- ❑ Diabetes Mellitus (DM)

9.During the pandemic, did you experience symptoms specific to COVID-19?

- ❑ YES
- ❑ NO

10.Did you have a PCR test confirming SARS-CoV-2 infection?

- ❑ YES
- ❑ NO

11.What type of illness did you have?

- ❑ Mild - asymptomatic or with minimal symptoms: sneezing, runny nose, conjunctival irritation, nasal congestion, no fever
- ❑ Moderate - cough, fever, chills, joint/muscle pain, digestive issues, chest pain, breathing difficulty, severe fatigue, loss of appetite
- ❑ Severe - requiring intubation and mechanical ventilation, stroke, myocardial infarction, pericarditis, etc., with ICU admission

12. Do you believe that increased mortality in patients with chronic conditions is directly related to SARS-CoV-2 infection?

- ❑ YES, due to aggravation of the underlying condition
- ❑ NO, it is unrelated to chronic disease status
- ❑ Mortality rates remained unchanged compared to the pre-pandemic period

13.In your opinion, death in a patient with at least one of these five chronic conditions who also contracts SARS-CoV-2 occurs due to:

- ❑ Treatable causes
- ❑ Untreatable causes

III. ACCESSIBILITY OF MEDICAL SERVICES

14.At the time of completing this questionnaire, which medical services were easier to access?

- ❑ Private medical services
- ❑ Public medical services

15.Did you attempt to make an appointment at an outpatient clinic/hospital/territorial polyclinic/individual medical office during the COVID-19 pandemic?

- ❑ YES
- ❑ NO

16.When did you receive the requested appointment/service?

- ❑ On the same day, within a few hours
- ❑ The next day
- ❑ After 1 day, but less than 7 days
- ❑ After more than 7 days, but less than 30 days
- ❑ After more than 30 days

17.To what extent do you feel this service was accessible during the health crisis?

- ❑ Inaccessible
- ❑ Difficult to access
- ❑ Easy to access

18.How did you obtain the appointment?

- ❑ By phone
- ❑ Online scheduling
- ❑ Emergency consultation in the ER, as I was unable to get an appointment

19.During the health crisis, were your appointments delayed?

- ❑ Never
- ❑ Occasionally
- ❑ Frequently
- ❑ Continuously delayed

20.Approximately how many appointments were delayed during the health crisis?

- ❑ None
- ❑ 1 appointment
- ❑ 1–3 appointments
- ❑ 3–5 appointments
- ❑ More than 5 appointments

21.Do you believe these delays in seeing a specialist worsened your health condition?

- ❑ YES
- ❑ NO

22.During the health crisis, do you believe the accessibility of medical consultations/treatments:

- ❑ Decreased
- ❑ Increased
- ❑ Remained the same

23.How do you think digitalization affected medical service accessibility during the COVID-19 pandemic?

- ❑ It improved
- ❑ It worsened
- ❑ It remained the same

24.Was it easier or more difficult than before the pandemic to obtain an online consultation appointment?

- ❑ Easier during the pandemic
- ❑ More difficult during the pandemic

25.What do you think were the causes of these delays?

- ❑ A higher number of hospital/clinic visits compared to before the pandemic
- ❑ Shortage of specialized medical personnel in Romanian hospitals
- ❑ Lack of protective medical equipment (FFP2 masks, gowns, face shields, etc.)
- ❑ Reduced number of hospital beds due to reorganization for isolation units

- ❑ Lack of specialized medical equipment

26.Do you think that the organizational changes required by the evolution of the SARS-CoV-2 pandemic led to decreased accessibility of medical services?

- ❑ YES
- ❑ NO

27.What type of service was most accessible for you?

- ❑ Outpatient consultation
- ❑ Emergency consultation in the ER
- ❑ Hospitalization for a scheduled intervention
- ❑ Day hospitalization

28.Would you recommend that your relatives/friends access medical services in Romania instead of abroad?

- ❑ Yes, I would recommend it
- ❑ No, I would never recommend it
- ❑ Only in cases of extreme necessity

29.How would you rate your health status after hospital discharge/specialty treatment/outpatient consultation?

- ❑ My health improved
- ❑ My health deteriorated
- ❑ My health remained unchanged

30.On a scale from 1 to 5, how would you rate the quality of nursing care during the pandemic?

- ❑ 1
- ❑ 2
- ❑ 3
- ❑ 4
- ❑ 5

THANK YOU FOR YOUR TIME!

Prepared by:

Drd. Despina Paula Andrei
Principal Investigator
Carol Davila University of Medicine and Pharmacy
